# Dopa-responsive dystonia caused by tyrosine hydroxylase deficiency: Three cases report and literature review

**DOI:** 10.1097/MD.0000000000021753

**Published:** 2020-08-14

**Authors:** Han-Yu Dong, Jun-Yan Feng, Xiao-Jing Yue, Ling Shan, Fei-Yong Jia

**Affiliations:** Department of Developmental and Behavioral Pediatrics, The First Hospital of Jilin University, Changchun, China.

**Keywords:** dopa-responsive dystonia, Segawa, tyrosine hydroxylase deficiency, autosomal recessive, compound heterozygous

## Abstract

**Rational:**

Tyrosine hydroxylase deficiency (THD) is a rare cause of dopa-responsive dystonia (DRD). Although the symptoms of DRD may be improved by treatment with L-dopa, the low morbidity of THD can lead to its misdiagnosis. Thus, it is important for physicians to be aware of THD as a cause of DRD.

**Patient concerns:**

We report 3 cases of THD. A 5-year-old boy with DRD was diagnosed with THD and found to have compound heterozygous mutations of the TH gene, including TH:c.647G>C from his mother and TH:c.646G>A from his father. Two female siblings also were found to have TH:c.698G>A from their mother and TH:c.710T>C from their father. The younger daughter, at age 3.5 years, was diagnosed with DRD caused by THD, and then the diagnosis of the older daughter, at age 11 years, was changed from cerebral palsy to DRD caused by THD.

**Diagnosis:**

The diagnosis of dopa-responsive dystonia caused by tyrosine hydroxylase deficiency was determined by whole exome sequencing.

**Intervention:**

They all treated with low dose levodopa and benserazide tablets.

**Outcomes:**

The boy had a very good therapeutic effect, and he could walk very well by the second day of treatment. The younger sister of the siblings had a partial therapeutic effect, but her elder sister was only little effective with a milder improvement of dystonia and improvement of myodynamia.

**Conclusion:**

The characteristics of THD are heterogeneous, and its phenotypes are classified as type A or type B according to increasing severity. Generally, L-dopa has a good therapeutic effect in cases with type A phenotypes. We reviewed 87 cases of reported in the literature and found that c.698G>A and c.707T>C are hot spot mutations. Changes on cerebral magnetic resonance imaging were nonspecific. Analysis of neurotransmitter levels in cerebrospinal fluid is an invasive means of achieving a biochemical diagnosis.

## 1 Introduction

Dopa-responsive dystonia (DRD), originally known as Segawa Syndrome after the Japanese physician who first reported it,^[1]^ was so named by Nygaard et al in 1988^[2]^ and is a rare condition of autosomal inherited dyskinesia characterized by uncontrolled muscle movement. The pathogenesis of DRD most commonly involves GTP cyclohydrolase-1(GCH-1) gene variants, but mutations of the tyrosine hydroxylase (TH) gene have also been identified in rare cases. A defect in the TH gene leads to tyrosine hydroxylase deficiency (THD), a condition in which the process by which tyrosine is converted to dopamine is blocked, which results in reduced production of catecholamines and leads to the clinical symptoms of DRD.

Due to its low morbidity, awareness of THD can be low, leading to its misdiagnosis. Here we report 3 cases of DRD caused by THD in which 4 genetic mutations were identified and also present a review of THD-related DRD cases found in the current literature. The aim of this report is to improve awareness of THD in order to promote early intervention and, thus, better patient outcomes.

## 2 Case report

### 2.1 Case 1

A 5-year-old boy presented with unsteady walking and limb tremor that had persisted for nearly 4 years. He was informed consent for publication of the case. He was second child of his mother, and he was born at full-term via cesarean section, weighing 3.8 kg. His parents felt that he had low sucking power during his neonatal period. He met the infant milestones of rolling over at 4 months, sitting unassisted at 6.5 months, and crawling at 8.5 months. However, he could not walk at 12 months and displayed tremor in both lower limbs. At age 2 years, his arms began to jitter and was unable to stably hold objects in his hands. He also had hypodynamia with very obvious diurnal fluctuation. His parents were healthy, and he had a healthy 12-year-old brother. The patient had no positive family history of DRD. His intelligence was normal, but he experienced dysarthria and muscular hypertonia characterized as cogwheel rigidity.

Routine biochemistry tests for hepatic and renal function, ceruloplasmin, blood lactate, blood ammonia, thyroid function, and chromosomes were all normal. The results of 24-hour electroencephalography (EEG) monitoring were normal. Findings on cerebral magnetic resonance imaging (MRI) and magnetic resonance angiography (MRA) were normal.

High-throughput sequencing (Running Gene Inc., Illumina NovaSeq) for whole exome sequencing revealed that the patient had inherited the TH variant c.647 G>C (p.G216A) from his mother. This mutation was not recorded in the Exome Sequencing Project Database, Ensembl Project Database, or Exome Aggregation Consortium Database. The patient has also inherited the TH variant c.646G>A (p.G216S) from his father. This mutation is a known missense mutation, for which the mutation rate is low. However, pathogenicity has been reported. Sangers DNA sequencing confirmed that these 2 mutations were from his mother and father, respectively, and constitute a complex heterozygotic pattern.

The patient was treated with levodopa and benserazide hydrochloride tablets at a dose of 3.5 mg/(kg/d), and a very good therapeutic effect was observed. He could walk very well by the second day of treatment. Therefore, his genetic and clinical diagnoses were DRD caused by THD.

### 2.2 Cases 2 and 3

Cases 2 and 3 were siblings. They were all informed consent for publication of their cases. Case 2 was the younger sister with an age of 3.5 years at presentation. She had shown delayed motor functioning after birth, and her parents reported that her motor function had regressed in the past year. She was the third child of her mother and born at full-term via cesarean section with a birth weight of 3.5 kg. She could roll over at 5 months but was seen by a doctor when she could not sit at 6 months. She was given a diagnosis of “developmental delay” and began rehabilitation therapy, but she showed no obvious improvement. Her parents were healthy, and she had an older brother with congenital heart disease, involving a ventricular septal defect, who passed away at age 5.

The patient could not speak, and her comprehension also seemed poor. She had level 4 myodynamia and dystonia, and these symptoms showed diurnal fluctuation. Routine biochemistry tests for hepatic and renal function as well as tandem mass analysis of a dried blood sample showed normal results. The results of 24 hour EEG monitoring also were normal, and MRI showed some punctiform abnormal signals.

High-throughput sequencing (Running Gene Inc., Illumina NovaSeq) for whole exome sequencing revealed that the patient had inherited the TH variant c.698G>A(p.R233H) from her mother. This mutation is a known missense mutation, for which the mutation rate is low and pathogenicity has been reported. She also inherited the TH variant c.710T>C(p.I237T) from her father, and this mutation was not recorded in the human gene mutation database. Thus, the pathogenicity is unclear.

The patient was treated with levodopa and benserazide tablets (3.5 mg/kg/d), and a partial therapeutic effect was observed. With this treatment, she was able to sit by herself, but still could not walk or speak. Unfortunately, whole exosome sequencing for this patient was not processed for protein function prediction, and therefore, we cannot confirm that the identified gene mutations lead to impaired TH protein function. Thus, her genetic diagnosis was unclear, but her clinical diagnosis was DRD caused by THD.

Case 3 was the 11-year-old sister of case 2. She had experienced development delay after birth and received a diagnosis of cerebral palsy. She was unable to roll over or sit unassisted, and she had no ability to perform activities of daily living. She had level 3 myodynamia and poor comprehension and expression abilities. Based on her younger sisters diagnosis with THD, we questioned whether she might also have the same diagnosis. Whole exome sequencing revealed the same 2 TH mutations as in the younger sister. Treatment with levodopa and benserazide tablets (3.5 mg/kg/d) was only partially effective with mild improvement of dystonia and improvement of myodynamia from level 2 to level 3, but no other changes. We changed her clinical diagnosis from cerebral palsy to DRD caused by THD.

Ethical review: The 2 families were informed and provided written consent. The ethics committee of our hospital approved this study.

## 3 Discussion

### 3.1 Overview of DRD

**Figure 1:**
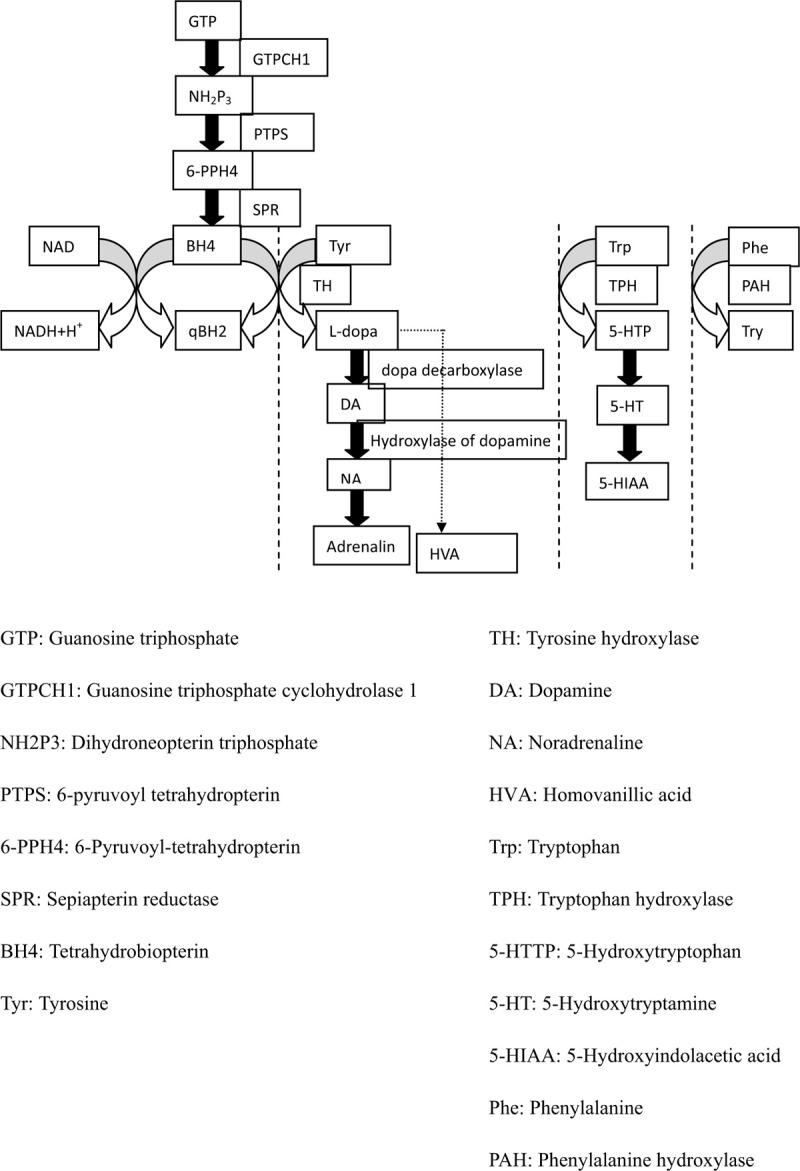
Metabolism of BH4, aromatic amino acids, catecholamine, and serotonin, which illustrate the pathogenesis of DRD and THD.

DRD is an autosomal inheritance movement disorder that occurs in children and adolescents and is characterized by progressive dystonia with diurnal fluctuation and shows a dramatic response to treatment with L-dopa. The disease predominantly affects females, and female patients typically have more severe symptom than male patients. The prevalence of DRD is reported to be 0.5 to 1.0 per million.^[3]^ The established pathogenesis involves mutations in the genes that encode enzymes responsible for dopamine or tetrahydrobiopterin (BH4) biosynthesis (Fig. 1).

The most commonly mutated gene in DRD cases is *GCH1*, which encodes the GCH1 protein, and this mutation occurs via autosomal dominant inheritance. *GCH1* is located on chromosome 14q22.1 to 22.2 and composed of 6 exons.^[4]^ GCH1 deficiency affects BH4 synthesis, which causes the metabolic pathways of aromatic amino acids to be blocked, leading to deficiencies in catecholamines (including dopamine, noradrenaline, and adrenalin) and 5-hydroxytryptamine as well as their metabolites, including homovanillic acid (HVA) and 5-hydroxyindolacetic acid (5-HIAA). These deficiencies lead to the classic clinical symptoms of DRD.

Mutation of the TH gene causing THD is an extremely rare pathogenesis of DRD. THD shows autosomal recessive inheritance and affects the rate-limiting step of catecholamine synthesis. The TH gene is located on chromosome 11p15 and composed of 14 exons.^[5]^ Because TH mutation affects the biosynthesis of only catecholamines and not 5-TH, the concentration of 5-HIAA—the metabolite of 5-TH—is normal. This can be an identifying characteristic of DRD caused by THD as opposed to GCH1 mutation.

### 3.2 Clinical characteristics of THD

THD presents a wide spectrum of clinical phenotypes,^[6]^ with great variation in symptoms. Hypokinesia, rigidity, dystonia, oculogyric crisis, salivation, and excessive sweating are specific symptoms of dopamine deficiency, and nonspecific symptoms include encephalopathy, psychomotor retardation, limb hypertonia with truncal hypotonia, and pyramidal tract dysfunction. Tremor and other forms of extrapyramidal track dysfunction are symptoms of catecholamine deficiency. The effects of THD can vary from mild DRD to lethal encephalopathy, and the specific phenotypes are classified as type A or type B clinically. Type A phenotypes correspond to mild progressive dystonia with an onset in infancy or childhood and usually more sensitive to treatment with L-dopa. Type B phenotypes have more severe symptoms with onset in the neonatal period or early infancy and show a moderate or poor response to treatment with L-dopa.

We reviewed all cases of THD reported through 2018 and have summarized the features of the 87 cases found.^[7–44]^ The age at onset ranged from the neonatal period to 18 years of age, and the average onset age was 13.4 months. The clinical features reported for 75 cases are described in Table 1. The most common symptoms were hypokinesia (54/75), truncal hypotonia (51/75), and dystonia (43/75). Our case 1 had hypokinesia, dystonia, tremor, dysarthria, and rigidity with a phenotype categorized as type A, whereas our cases 2 and 3 had encephalopathy, psychomotor retardation, and dystonia with a phenotype categorized as type B. Among the previously reported case, 38.7% had obvious diurnal fluctuation of symptoms, which has been noted in the literature^[13]^ and was observed in our case 1. Among 43 cases for which cerebral MRI was performed, abnormal findings were reported for only 9 cases, including bilateral widening of the frontotemporal extracerebral space, ventriculomegaly, and other nonspecific changes, indicating that the pathogenesis of brain connectivity impairment in most cases was caused by dopamine deficiency rather than a defect in cerebral structure.^[21]^ MRI in our case 2 showed some punctiform abnormal signals, which were not responsible for the patients symptoms, and MRI appeared normal for our case 1.

**Table 1:**
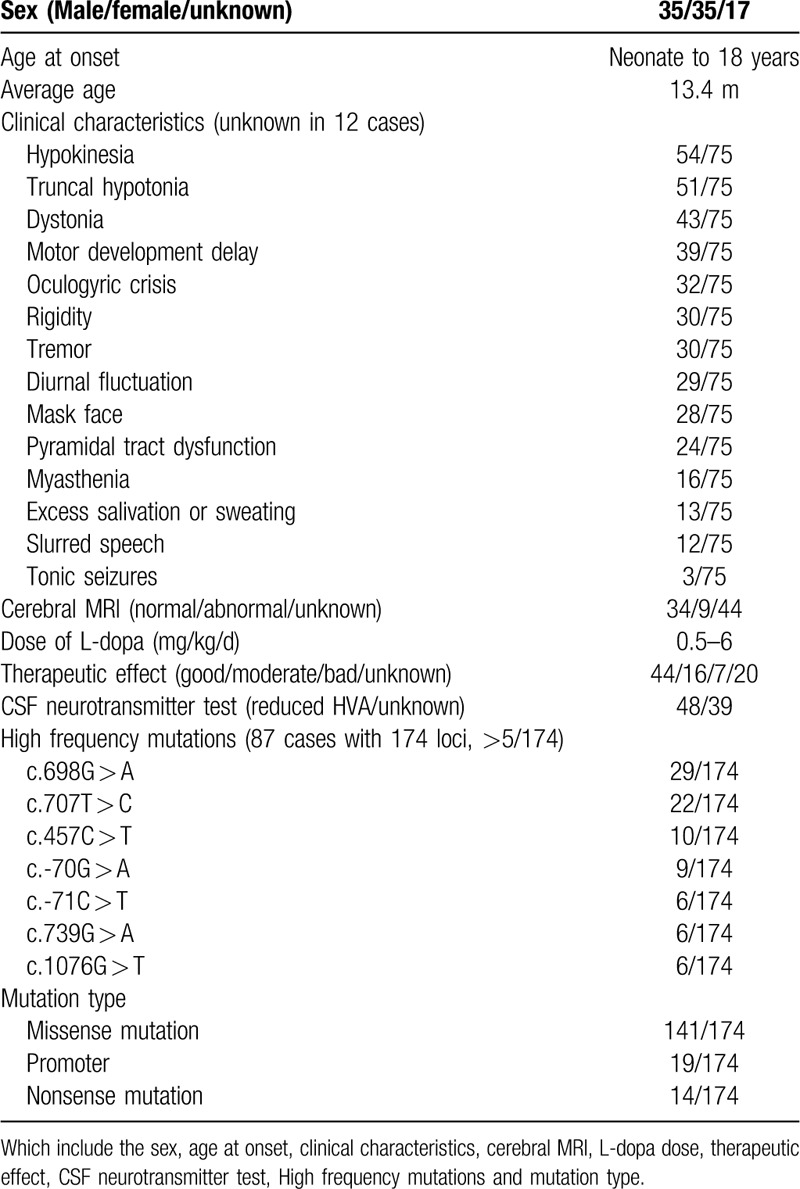
Summary of THD cases reported in the literature through 2018.

### 3.3 Treatment

L-dopa is considered a miracle drug for THD, and usually, even at a low dose, L-dopa has a very good therapeutic effect. However, in some cases with type B phenotypes, L-dopa has no effect or even some adverse side-effects. In the reviewed literature, the dosage range for L-dopa was 0.5 to 6 mg/kg/d, and in our 3 cases, the patients were given a dose of 3.5 mg/kg/d. The therapeutic effect of L-dopa was reported for 67 of the 87 cases reported in the literature, and among these the therapeutic effect was good in 44 cases, moderate in 16 cases, and poor or absent in 7 cases. Although we cannot surmise the phenotype of each previously reported case based on the available information, the effectiveness of L-dopa likely corresponded to the distribution of type A and type B phenotypes. Additional research is needed to confirm this relationship.

In our case 1, L-dopa treatment had a good effect. The treatment effect in case 2 was moderate, and in case 3, L-dopa treatment had almost no effect. No adverse side effects were observed in any of the cases. The reasons why some cases with type B phenotypes show no response to L-dopa remain unclear. In some cases, the dosage of L-dopa may have been insufficient or intestinal resorption of L-dopa may have been inadequate.^[29]^ Thus, it may be practicable to increase the dose of L-dopa for our cases 2 and 3.

Some other drugs have been used for the treatment of THD as assistant drugs. Benzhexol is a central choline blocker that is used to control tremor and other extrapyramidal system symptoms. Benserazide and carbidopa are peripheral dopamine decarboxylase inhibitors that increase dopamine levels. Selegiline is a type of monoamine oxidase inhibitor that prolongs the duration of dopamine action. Biperiden is also used to control tremor and other extrapyramidal system symptoms. We recommend the use of several types of these drugs in combination, and in China, the combination of L-dopa and benserazide is commonly used and found to have a good effect.

### 3.4 Cerebrospinal fluid (CSF) neurotransmitter analysis

Lumbar puncture is an invasive means for biochemical diagnosis. Among the 87 reported cases of DRD due to THD, 48 cases had undergone CSF neurotransmitter analysis, and all 48 patients had a reduced level of HVA and normal level of 5-HIAA. The concentration of HVA in CSF can provide an estimate of the activity of TH, and thus, offers a method for evaluating the therapeutic effect of L-dopa. Moreover, the HVA/5-HIAA ratio was reported to show a positive correlation with the age of onset of THD and may be useful for predicting the effect of L-dopa and the patients overall prognosis.^[35]^ Unfortunately, lumbar puncture for CSF neurotransmitter analysis was not performed in our 3 cases, because of the invasiveness of the procedure. However, in the absence of genetic analysis, CSF analysis would be useful for confirming whether DRD is caused by THD or another gene deficiency that affects BH4 biosynthesis.

### 3.5 Molecular analysis

The human TH gene (mRNA type 1) contains 14 exons with an open reading frame of 1491 bp and encodes a protein with 497 amino acids that is highly conserved among various species.^[6]^ Homozygous mutations or compound heterozygous mutations of the TH gene cause THD. To date, 66 pathogenic TH gene mutations have been reported, including the novel mutation c.647G>C identified in our case 1 and c.710T>C identified in our cases 2 and 3. Among these 66 mutations, there are 14 nonsense mutation, which translate into termination codons. Nineteen of these mutations are in promoters and thus affect the initiation of protein translation. This suggests that if THD is suspected, TH gene analysis is appropriate for not only all exons but also the promoter and intron, which may also affect the expression of the protein. The TH mutations with the highest frequencies are listed in Table 1. No phenotype and genotype correlations have been made with THD, and thus, we summarized the hot spot mutations in the reported cases. Altogether 174 mutations were reported among the 87 patients. The hot spot mutations were c.698G>A (29/174) and c.707T>C (22/174). Among the 67 cases for which a therapeutic response was observed, 23 cases had type B phenotypes. Among 21 cases with the c.698G>A variant, 7 cases had type B phenotypes. Five cases had the c.707T>C variant, and they all had type B phenotypes. It seems that c.707T>C may preferentially exist in type B as opposed to type A cases, but further research is needed to determine this. Overall, at present it seems that the genotype cannot predict the phenotype, but perhaps this will be possible with further study of the function of the TH protein.

## 4 Conclusion

THD is a rare and severe neurometabolic disorder. However, it is often treatable, and thus, early and accurate diagnosis are critical. Diagnosis of THD relies on the clinical symptoms and experimental treatment with L-dopa. Of the 2 categories of phenotypes, which are based on the severity of symptoms, cases with the less severe type A phenotypes generally show a good therapeutic response to L-dopa. Neurotransmitter analysis and genetic testing for TH gene mutations are also important. Among the THD cases reported to date, the hot point missense mutations were c.698G>A and c.707T>C, and no relationship has been found between genotype and phenotype in THD cases.

## Author contributions

**Conceptualization:** Xiaojing Yue.

**Resources:** Junyan Feng.

**Writing – original draft:** Hanyu Dong, Ling Shan.

**Writing – review & editing:** Hanyu Dong, Feiyong Jia.
